# Dystrophic Cardiomyopathy: Complex Pathobiological Processes to Generate Clinical Phenotype

**DOI:** 10.3390/jcdd4030014

**Published:** 2017-09-08

**Authors:** Takeshi Tsuda, Kristi K. Fitzgerald

**Affiliations:** Nemours Cardiac Center, Nemours/Alfred I. duPont Hospital for Children, Wilmington, 1600 Rockland Rd, DE 19803, USA; kristi.fitzgerald@nemours.org

**Keywords:** dystrophinopathies, cardiomyopathy, dystrophin-glycoprotein complex (DGC), epigenetic, duchenne muscular dystrophy (DMD), genotype-phenotype correlation

## Abstract

Duchenne muscular dystrophy (DMD), Becker muscular dystrophy (BMD), and X-linked dilated cardiomyopathy (XL-DCM) consist of a unique clinical entity, the dystrophinopathies, which are due to variable mutations in the dystrophin gene. Dilated cardiomyopathy (DCM) is a common complication of dystrophinopathies, but the onset, progression, and severity of heart disease differ among these subgroups. Extensive molecular genetic studies have been conducted to assess genotype-phenotype correlation in DMD, BMD, and XL-DCM to understand the underlying mechanisms of these diseases, but the results are not always conclusive, suggesting the involvement of complex multi-layers of pathological processes that generate the final clinical phenotype. Dystrophin protein is a part of dystrophin-glycoprotein complex (DGC) that is localized in skeletal muscles, myocardium, smooth muscles, and neuronal tissues. Diversity of cardiac phenotype in dystrophinopathies suggests multiple layers of pathogenetic mechanisms in forming dystrophic cardiomyopathy. In this review article, we review the complex molecular interactions involving the pathogenesis of dystrophic cardiomyopathy, including primary gene mutations and loss of structural integrity, secondary cellular responses, and certain epigenetic and other factors that modulate gene expressions. Involvement of epigenetic gene regulation appears to lead to specific cardiac phenotypes in dystrophic hearts.

## 1. Introduction A

### 1.1. Dystrophinopathies

Dystrophinopathies are caused by genetic mutations in the dystrophin gene that include Duchenne muscular dystrophy (DMD), Becker muscular dystrophy (BMD), and X-linked dilated cardiomyopathy (XL-DCM) [1,2]. Dystrophin is a part of a protein complex called dystrophin-glycoprotein complex (DGC) that provides a mechanical link between contractile apparatus and extracellular matrix through the cell membrane [3]. Deficiency of dystrophin in the myocyte results in loss of physical integrity of muscle cells and causes contraction-induced muscle degeneration [4]. All three entities are inherited as an X-linked recessive trait; de novo mutations occur in one third of cases. Some female carriers develop similar but milder clinical phenotypes [5]. Patients with DMD show severe progressive muscle weakness and wasting, and usually lose independent ambulation by the age of 12 without corticosteroid treatment [6], although there is a substantial age variation in wheelchair dependence [7]. They become progressively disabled with multiple organ involvement including respiratory system issues, neuromuscular scoliosis, and cardiomyopathy [8,9,10]. Central nervous system (CNS) involvement is seen frequently in DMD, including cognitive dysfunction, neuropsychological problems (anxiety, depression, and emotional disturbance), and neurobehavioral abnormalities (autism spectrum, attention deficit hyper activity disorder, and obsessive compulsive disorder) [11,12]. The prevalence of intellectual disability in DMD patients and general population is reported to be 20.9% and 3%, respectively [13]. The average IQ of a boy with DMD is 85, which corresponds to −1 standard deviation (SD) in normal control population [14]. Cardiomyopathy is usually seen in the middle to late teens, but the clinical presentation is insidious, as the patients are already wheelchair-bound and are not required to perform increased cardiac workload [15]. Nigro et al. estimated that the overall incidence of latent DCM is 25% by age six years and 59% by age 10 years in DMD patients [16]. Patients with BMD have a milder clinical phenotype of DMD, and most patients remain ambulatory until the fourth or fifth decade of life. Skeletal muscle involvement is milder than in DMD. Cardiac complications commonly do not become clinically evident until the fourth decade of life, but the cardiac function deteriorates rapidly to cause fatal heart failure once myopathic changes occur [17,18,19,20,21,22]. The most common cause of death in BMD is DCM and associated heart failure [23]. Rapidly progressive DCM with symptomatic congestive heart failure has been reported as a rare occurrence in younger adolescents with BMD [24,25]. XL-DCM is a familial DCM with very little or no skeletal muscle involvement [26,27,28]. The onset of DCM in XL-DCM is during the late teens to early twenties with progressive deterioration or mid-adult onset of insidious process [27]. All these distinct clinical presentations derive from mutations in the same dystrophin gene. Thus, dystrophinopathies are an excellent human model to study how genetic mutation generates phenotype and how secondary intermediate factors are involved in modifying final clinical presentation.

### 1.2. Cardiac Phenotype of Dystrophinopathies

Myocardial involvement is inevitable in DMD patients, as dystrophin serves the same biological role in cardiomyocytes as in skeletal muscle cells [2]. Pathological alteration of ventricular myocardium in DMD is heterogeneous and probably a result of a combined consequence of myocardial wasting (atrophy) [29,30] and secondary myocardial remodeling (ventricular dilatation and fibrosis) [31]. The remodeling process occurs in combination with secondary fatty infiltration and fibrosis within the myocardial tissue [32]. Although DMD cardiomyopathy is conventionally addressed as DCM, the affected hearts do not always show ventricular enlargement [33]; in fact, some show small-to-normal heart size with significantly thin ventricular wall [34,35]. Thin ventricles are just as disadvantageous as dilated ventricles because of their increased wall stress without compensatory ventricular hypertrophy. 

Clinical manifestations of dystrophic DCM include congestive heart failure due to ventricular dysfunction, arrhythmia, and sudden cardiac death [15,36,37]. In the latter part of this review, we will discuss in detail the cellular and molecular mechanisms of failing dystrophic hearts. Because of the inability of DMD patients to ambulate at the time of diagnosis, common symptoms of congestive heart failure are not evident. Instead, patients tend to complain nonspecific symptoms including sleep disturbance, nausea, anorexia, cough and increased secretion, and weight loss [38]. Natural history of rhythm abnormality has not been well documented in the literature [39]. Myocardial fibrosis usually precedes LV dysfunction, and its presence serves as an independent indicator of LV dysfunction [32,40]. In the study by Chiang et al., arrhythmias occurred in 44% of DMD patients and 57% of BMD patients, and were significantly correlated with decrease in cardiac function [41]. Clinically significant tachyarrhythmias, supraventricular tachycardia (SVT) and ventricular tachycardia (VT), were seen in 10% and 25% of DMD and BMD patients, respectively [41]. Sinus tachycardia is commonly seen in teenage DMD patients, but underlying pathology is not entirely clear. Sinus tachycardia may represent an early sign of ventricular dysfunction in DMD [42], but autonomic dysfunction is, in part, responsible for inappropriately elevated heart rate [43,44]. Persistently elevated heart rate will increase myocardial oxygen demand, and thus may impose further negative impact on dystrophic myocardium [45]. Sporadic cases of sudden death have been reported in DMD patients, but their relationship to ventricular arrhythmia has not been well established [46]. Ventricular arrhythmias and sudden death may play a similar role in DMD as in other forms of non-ischemic DCM [47].

There is significant variation in the onset, progression, and severity of DCM in these three dystrophinopathies that share mutations of the same dystrophin gene [1]. In these three dystrophinopathies, what determines the clinical phenotype and the onset and severity of cardiomyopathy? Do certain genotypes correspond to specific phenotypes? To understand these questions, complex molecular and cellular mechanisms should be delineated. In this review article, we discuss multiple layers of pathologic processes seen in the dystrophic hearts, including primary structural fragility, secondary cellular responses, and specific epigenetic and other factors that modulate gene expressions that determine the final clinical phenotype. 

## 2. Molecular Genetics of Dystrophinopathies

### 2.1. Dystrophin-Glycoproein Commplex and Its Molecular Structure

**Figure 1:**
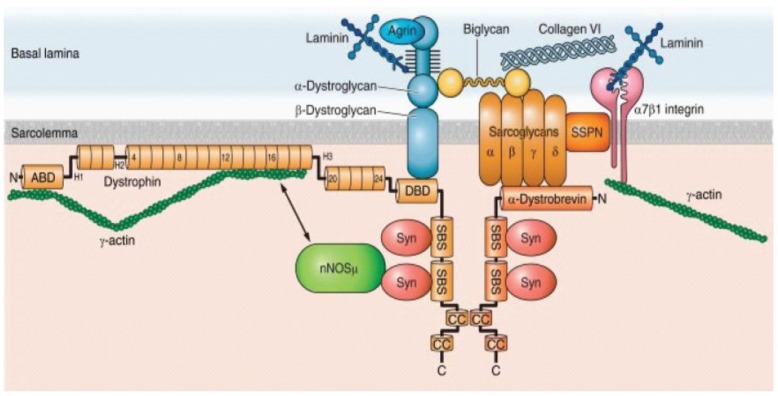
The dystrophin glycoprotein complex (DGC) in a skeletal muscle cell. Shown are the interactions among core components of the DGC, the extracellular matrix, and nNOS. Numbers in dystrophin indicate hinge regions (H1, H2, etc.) and spectrin-like repeat domains (4, 8, 12, etc.). However, nNOS is not associated with DGC in a cardiomyocyte (see the text for detail). nNOS, neuronal nitric oxide synthase; Syn, syntrophin; SSPN, sarcospan; ABD, actin binding domain; DBD, dystroglycan binding domain; SBS, syntrophin binding site; CC, coiled-coil domain; N, amino terminus; C, carboxy terminus. Obtained from Allen DG, Whitehead NP, and Froehner SC, “Absence of Dystrophin Disrupts Skeletal Muscle Signaling: Roles of Ca^2+^, Reactive Oxygen Species, and Nitric Oxide in the Development of Muscular Dystrophy” *Physiol. Rev.* **2016**, *96*, 253–305, under copyright agreement.

The human dystrophin gene, *DMD*, is a massive gene spanning 2.2 Mb of DNA consisting of 79 exons with three main promoters producing full-length dystrophin and at least four promoters transcribing shortened dystrophin isoforms with developmental and tissue-dependent regulation. *DMD* encodes the dystrophin protein, which is a membrane-associated protein [1,48,49]. Dystrophin consists of an *N*-terminal actin-binding domain, followed by a large central rod domain including 24 spectrin-like homologous repeat units that form an α-helical structure followed by a cysteine-rich domain that binds dystroglycan, a component of the extracellular matrix, and then a *C*-terminal domain [50]. A primary role of dystrophin is to form a mechanical link between cytoskeletal actin and the extracellular matrix, where dystrophin is an important component of the large oligomeric complex of sarcolemmal glycoproteins that form DGC. (Figure 1) Specifically, the *N*-terminal and rod domains of dystrophin associate with the cytoplasmic γ-actin filaments, and the cysteine-rich domain binds directly to the β-subunit of the dystroglycan complex. In the absence of dystrophin, the muscle membrane is susceptible to damage and deterioration. In the skeletal muscle, over time, cycles of degeneration and regeneration result in fibrosis and fatty replacement of muscle tissue [2]. 

The DGC has been studied in human and animal cardiomyocytes [51,52,53]. The DGC in human cardiac muscle has a costameric distribution, and has been shown to be present in the T-tubules and intercalated disks [51]. In that study, the proteins of the DGC colocalized with each other and with the proteins of the vinculin-talin-integrin system and all proteins were in the region of the sarcolemma over the I band [51]. The results suggest a role for the cardiac DGC in transduction of the mechanical force to the extracellular matrix in cardiac muscle. These results have been shown in animal models as well, where there was a striking difference of the distribution of the DGC between cardiac and skeletal muscle in that the DGC localizes to the regions where T-tubules are distributed in cardiac muscle but not in skeletal muscle [53].

To date, more than 5000 pathogenic variants have been identified in *DMD* [54]. The mutation types vary depending upon the specific clinical cohort of DMD and BMD patients. However, in general, 60–70% have exonic deletions; 5–10% have exonic duplications; and 25–30% of DMD patients, and 10–20% of BMD patients have single nucleotide variants including small deletions or insertions, single base changes, and splice site changes [55,56,57,58,59]. There are two recombination hot spots with partial deletion and duplication clusters: one including exons 2–20, and the another more distal including exons 44–53 [60]. Duplications cluster towards the 5′ end of the gene with duplication of exon 2 being the single most-common duplication [59]. 

### 2.2. Spatial Expression of Dystrophin

Dystrophin has seven tissue-specific promoters encoding full-length and truncated isoforms of the protein. Full-length dystrophin is expressed in all muscle types including skeletal, smooth, and cardiac myocytes, and all muscle fiber types. Immunofluorescence and electron microscopy of skeletal muscle indicate dystrophin is localized to the membrane or sub-membrane and is undetectable in transverse (T)-tubules or intracellular structures [61]. The primary isoform found in skeletal muscle and responsible for the DMD and BMD phenotype is Dp427m. This isoform is expressed in skeletal and cardiac muscle (Byers TJ, Leiden Muscular Dystrophy Pages: Dystrophin isoforms. http://www.dmd.nl/isoforms.html: last modified on 5 March 2006). 

In cardiomyocytes, the dystrophin isoforms Dp427 and Dp71 are both expressed; however, in skeletal muscle, only Dp427 is present. In contrast to skeletal muscle, dystrophin is located in the cardiac T-tubule [62]. In a study using DMD-null mice in which both Dp427 and Dp71 were absent and using *mdx* mice in which Dp427 was absent but Dp71 was present [63], the authors showed that cardiomyopathy is caused predominantly by a deficiency of full-length dystrophin Dp427. In that study, Dp427 was located in the cardiac sarcolemma and the T-Tubules, and Dp71 was specifically located at the T-Tubules only [63]. 

Dystrophin isoforms are present in brain, although much less abundantly than in muscle, and they exhibit developmental, regional, and cell-type specificity [64]. Specifically, isoforms expressed in central nervous system (CNS) include Dp140 and Dp71 [64]. The brain-type promoter of dystrophin is highly specific to neurons, whereas the muscle-type promoter is active in a wide range of cell types including smooth muscle, glial cells, and neurons [65]. Areas of greatest expression include cerebellum, hippocampus, and cerebral neocortex. Areas of least expression include the basal ganglia and brainstem. The hippocampus and neocortex are directly involved in regulating emotions, memory, and cognitive processes [61]. 

In discussion of developmental expression of dystrophin, it is imperative to discuss utrophin, which is an autosomal homolog of dystrophin with significant sequence and protein structure similarity. In contrast to dystrophin, utrophin is ubiquitously expressed [66]. Utrophin expression occurs earlier than that of dystrophin in developing and regenerating skeletal muscle [67]. At birth and/or in mature skeletal muscle, utrophin is replaced by dystrophin at the sarcolemma, and utrophin expression is confined to the neuromuscular junction and vasculature [68]. In some myopathies, including the dystrophinopathies, utrophin is found at the sarcolemma [69]. 

Dystrophin is present early in development, is expressed in the neural tube and areas of the embryonic and postnatal neuroaxis, and may be involved in neurogenesis, neuronal migration, and cellular differentiation [64]. In the mature brain, dystrophin is expressed by specific regional neuronal subpopulations within proximal somadendritic microdomains associated with synaptic terminal membranes, and in adults, dystrophin modulates synaptic integrity, distinct forms of synaptic plasticity, and regional cellular signal integration [64]. Isoform Dp71 is the most abundant dystrophin transcript in brain and is undetectable in fully differentiated skeletal muscle [61], and Dp140 is found throughout the CNS and in the kidney [70]. Other isoforms include Dp116, which is expressed exclusively in adult peripheral nerve along with Schwann cell membrane, whereas full-length dystrophin and Dp71 are absent and Dp 260 is expressed in the retina [71] (Byers TJ, Leiden Muscular Dystrophy Pages: Dystrophin isoforms. (http://www.dmd.nl/isoforms.html: last modified on 5 March 2006).

### 2.3. Genotype-Phenotype Correlation

#### 2.3.1. Reading Frame Rule

Extensive studies related to the spectrum of phenotypes in the dystrophinopathies have been performed. In general, the phenotypic spectrum observed in the dystrophinopathies is related to the specific tissue and degree of dystrophin expression, which, in large part, is determined by the “reading frame rule” [72]. Originally defined in 1988 [72], the rule states pathogenic variants that do not alter the reading frame and thus allow translation of an internally truncated protein with functional *C*-terminus generally correlate with the milder BMD phenotype. Conversely, pathogenic variants that alter the reading frame and result in prematurely truncated dysfunctional dystrophin, leading to near complete or complete absence of dystrophin, result in the more severe DMD phenotype [72]. The genotype-phenotype correlation is accurate in about 91–92% of cases [54].

In BMD, the “reading frame rule” is less accurate. In a large Japanese cohort, 15% of patients with BMD caused by a deletion and 34% of patients with a duplication failed to adhere to the rule [56]. In another patient series, 30% of patients with a duplication with BMD failed to follow the reading frame rule [73]. Additionally, there are genotype correlations for the DCM phenotype in BMD. Specifically, the earliest age of onset of DCM (early 20s) is associated with mutation affecting the amino-terminal domain, out-of-frame mutations in the region of exons 45–49 result in DCM onset in the mid-20s, and deletions involving part of the rod domain and hinge 3 predict later-onset disease in the mid-40s [74]. 

Mutations causing XL-DCM or mild BMD with DCM are generally classified into four regions throughout the *DMD* gene: (1) the region of the muscle promoter to exon 1; (2) the region from exon 2–8 coding the actin binding domain; (3) the region from exon 45–55 (considered the hot-spot for mutations in the DMD gene that encodes the rod domain); and (4) the remaining region [75]. There are theories related to the mechanisms of pathogenicity in XL-DCM in which DCM occurs in the absence or near absence of skeletal myopathy. There are three full-length dystrophin isoforms: one expressed in skeletal and cardiac muscle, the second in the central nervous system, and the third in the cerebral Purkinje-cells. Pathogenic variants may alter transcriptional regulation of the *DMD* in different tissues. Pathogenic variants that affect the muscle isoform promoter (P_M_) and the first exon (E1) result in no cardiac muscle transcript. As a result, compensatory overexpression of two alternative promoters, brain (PB) and Purkinje (PP), that are typically only active in the brain are active in the skeletal muscle, allowing for dystrophin expression sufficient to prevent manifestation of skeletal muscle symptoms [76,77,78,79].

Another hypothesis regarding genotype-phenotype correlation in XL-DCM is the related cardiospecific stability of dystrophin and the interaction between dystrophin and its binding proteins. For example, in a family with XL-DCM with a missense mutation in exon 29, there was a decrease in dystrophin up to 20% of normal in skeletal and cardiac muscle; however, β- and δ-sarcoglycans were clearly decreased in sarcolemma of the cardiac muscle but not in the skeletal muscle tissue, showing that molecular changes in dystrophin yielding structural changes within the protein may disrupt dystrophin-associated proteins [80]. Missense mutation Lys18Asn in the *N*-terminal actin binding domain causes XL-DCM, although Singh et al. showed the mutation does not affect the protein’s overall secondary structure and function. However, the protein stability was decreased, protein unfolding was increased, and the protein structure was disrupted, indicating the stability and structure of dystrophin may be important in the pathogenesis of XL-DCM [81].

#### 2.3.2. Alternative Splicing and Exon Skipping

Careful correlation of clinical history and dystrophin molecular analysis is critical, as exceptions to the reading frame rule exist. Patients with in-frame mutations can present with DMD, and conversely, patients with out-of-frame mutations can have DMD. In some instances, the same mutation can produce both DMD and BMD phenotypes. For example, the relatively common out-of-frame deletion of exons 3–7 has been described in both DMD and BMD phenotypes depending upon whether the alternative translational initiation at the ATG in exon 8 is used [82,83]. Nonsense mutations are expected to result in premature protein termination and, therefore, the severe DMD phenotype. However, Flannigan et al. showed that 14% of nonsense mutations in *DMD* are associated with the BMD phenotype rather than DMD as predicted [84]. By analyzing the reading frame predicted by exons flanking those with the nonsense mutations, the authors showed evidence that the BMD phenotype is likely due to mutation-induced exon skipping in “in-frame” exons, where flanking exons would be predicted to maintain the reading frame when spliced together and thus continue protein translation [85]. Large in-frame deletions exceeding 36 exons are typically associated with a severe clinical phenotype of DMD [86,87], as are large in-frame deletions involving the 5′ region initiating at exon 3 or 4 and continuing into the rod domain [86,88,89,90]. Conversely, patients with a large in-frame deletion within the rod domain have been associated with a BMD phenotype [91].

## 3. Molecular Mechanisms of Dystrophic Hearts

Dystrophic hearts represent a unique clinical entity of mutations in *DMD* that cause variable abnormal cardiac phenotypes, including onset, progression, and severity of the heart disease. This entity serves as an excellent model to study how specific gene mutations generate cardiomyopathy and how secondary factors are involved in the pathogenesis [1]. Extensive investigations into the involvement of genetic factors have been undertaken to delineate the genotype-phenotype correlations of human dystrophinopathies [92,93,94]. A novel insight into the underlying molecular mechanisms of dystrophic hearts has also been generated by using animal models: dystrophin-deficient mice (*mdx* mice) and their variants (*mdx^2cv^*, *mdx^4cv^* and *mdx^5cv^*) [95], double mutant mouse models including utrophin-deficient *mdx* mice [96,97], and *mdx/myoD*-deficient double mutant mice [98]. These double knockouts have been accepted as more-accurate animal models to study cardiomyopathy in DMD, as *mdx* mice do not develop cardiomyopathy until later in life [96,97,98]. 

Pathogenesis of dystrophic cardiomyopathies consists of primary increased structural vulnerability to mechanical stress due to deficient cytoskeletal component [99] and secondary cellular responses [100,101]. The membrane instability and stretch-induced cardiomyocyte damage have been shown to be protected by using chemical-based membrane sealant, poloxamer, in isolated cardiomyocyte [102] and in dystrophin-deficient dog in vivo [103]. The tear of myocyte membrane, sarcolemma, with subsequent dysregulation of influx and efflux of ions triggers disruption of Ca^2+^ homeostasis and increased intracellular Ca^2+^ concentration; decreased nitric oxide (NO) production via nNOS; increased reactive oxygen species (ROS) and mitochondrial dysfunction; and induction of cell death pathways, apoptosis and necrosis, via mitochondrial signaling [100,101]. In addition to these events, certain epigenetic factors contribute to myocyte vulnerability and damage, as seen in other forms of heart failure [104,105]. Other contributing factors including telomere dysfunction [106,107], post-translational modification [108,109], and protein–protein interactions [110,111] may affect the phenotype. Dystrophic hearts are the result of multiple layers of pathology that accumulate to create a final cardiac phenotype.

### 3.1. Deficiency of Structural Integrity of DGC

A sequence of multiple molecular and cellular events is responsible for skeletal muscle degeneration and dystrophic heart [112]. The deficiency of DGC weakens the strong link between a contractile apparatus and extracellular matrix and, thus, increases the likelihood of contraction-induced microtear of sarcolemma [2,102,113]. In the skeletal muscle, DGC also forms a mechanically strong link between sarcolemma and costameric actin that generates structural integrity by supporting the alignment of myofibers [113]. However, structural fragility alone does not fully explain the onset and severity of muscle fiber impairment and death in dystrophinopathies. 

Microtear of the sarcolemma allows excessive influx of Ca^2+^ into the cell, resulting in cytosolic Ca^2+^ overload, which induces multiple downstream abnormalities [101]. As a second messenger, intracellular Ca^2+^ has many signaling roles ranging from cell death to muscle contraction [114]. Another second messenger disrupted in dystrophic hearts is nitric oxide (NO) because nNOS activity is considerably reduced in the absence of DGC [115]. Increase in reactive oxygen species (ROS) may be induced not only by exposure to mechanical stress [116] but also by increased intracellular Ca^2+^ and/or secondary mitochondrial dysfunction [100,117]. Mitochondrial dysfunction is induced by dysregulation of Ca^2+^ homeostasis, altered NO pathways, and increased ROS production, which may induce further pathological downstream pathways including abnormal energetics, ROS accumulation, and cell death [118]. 

### 3.2. Secondary Abnormal Cellular Responses in Myocytes

#### 3.2.1. Intracellular Ca^2+^ Increase

The biological role of Ca^2+^ signaling in dystrophic myocytes has been extensively reviewed [100,101,112,117]. Intracellular Ca^2+^ plays a principal role in excitation-contraction (E-C) coupling and signal transduction, through which increased intracellular Ca^2+^ induces detrimental contractile function and Ca^2+^-dependent downstream signaling pathways, respectively [114,119]. Increased intracellular Ca^2+^ in dystrophin deficiency is caused primarily by passive influx of Ca^2+^ by microtear of sarcolemma enhanced by muscle contractions [120,121]. In the dystrophic cardiomyocytes, intracellular Ca^2+^ overload occurs in response to stretch-induced microtears of sarcolemma and secondary enhancement of Ca^2+^ influx pathway [122]. Other mechanisms are known to contribute to intracellular Ca^2+^ overload, including stretch-activated channels (SACs) [123], mechano-sensitive transient receptor potential cation (TRPC) channels [124], Na-Ca^2+^-exchanger (NCX) [122], and voltage-gated Ca^2+^ channels (VGCC) or L-type channel [125]. A functional communication between VGCC and mitochondria plays an important role for metabolic function in healthy cardiomyocytes, which is compromised in *mdx* cells [125]. Intracellular Ca^2+^ sensitivity is also enhanced by cellular Ca^2+^ signal amplification mechanisms (Ca^2+^-induced Ca^2+^ response or CICR) [109]. Elevated intracellular Ca^2+^ in the heart leads to activation of numerous Ca^2+^-dependent pathways such as calpains (Ca^2+^-dependent proteinases, which degrade membranous proteins and contribute to necrosis) [126] and mitochondrial-mediated cell death [118,127].

#### 3.2.2. NO and nNOS Pathways

An important second messenger in cell physiology is nitric oxide (NO), which is regulated by NO synthase (NOS). Neuronal NOS (nNOS) and endothelial NOS (eNOS) are both constitutively expressed in muscle and heart and are activated by the Ca^2+^-dependent calmodulin [128]. NO plays an important physiological role in several pathways inside the skeletal muscle cells, including regulation of contractility, mitochondrial oxygen metabolism, glucose homeostasis, and blood flow [128,129]. Because nNOS is displaced in dystrophin-deficient skeletal muscle cells, nNOS activity levels are considerably lower in DMD patients and *mdx* mice, although nNOS deficiency is not due solely to lack of dystrophin in localizing the protein to the membrane as mRNA level of nNOS was also reduced in muscle biopsy of DMD patients and *mdx* mouse muscle [115]. In fact, in cardiomyocytes, nNOS is not only located at the sarcolemma [130], but also at the sarcoplasmic reticulum [131], mitochondria [132], and intercalated discs [133], where dystrophin is not expressed [134]. One proteomic analysis showed that nNOS does not interact with full length dystrophin in cardiomyocytes [135]. Thus, the deficiency in nNOS activity in cardiomyocytes in dystrophic hearts may primarily reflect defects in regulation rather than loss of binding to the DGC [136]. Stretch-dependent NO signaling has been shown to be impaired in dystrophin-deficient cardiomyocytes secondary to lack of AMPK activation, suggesting that DGC serves as a mechanosensor that regulates nNOS activity via AMPK signaling [137]. Reduced NO-cGMP signaling pathway may be a key contributor to DMD cardiopathogenesis, as a blockage of cGMP breakdown by sildenafil, a specific phosphodiesterase (PDE)-5 inhibitor, has reversed cardiac dysfunction in *mdx* mice [138] and prevented stress-induced cell death [139]. Other NO agonists have shown increased protection against developing dystrophic cardiomyopathy in *mdx* mice [136,140]. Deficient NO production in DMD patients and *mdx* mice also alters epigenetic regulation of chromatin changes via compromised histone modification [141]. However, a biological role of nNOS in the failing heart is not entirely clear, as overproduction of NO by enhanced nNOS activity counteracting a decrease in eNOS activity is demonstrated in human heart failure [142]. Further investigation will be necessary to determine the therapeutic approach to restore NO pathways. 

#### 3.2.3. Increased ROS and Mitochondrial Dysfunction

Mitochondria play an important role in energy metabolism and healthy muscle contraction. Mitochondria also function as a Ca^2+^ store, supplying and taking up Ca^2+^ to and from the cell [143,144]. Upon encountering excessive intracellular Ca^2+^, mitochondria produce ROS, which leads to cell death through necrosis and apoptosis by activating pathological signaling pathways [118]. In *mdx* mice, an antioxidant treatment reduced myocardial ROS, attenuated the myocardial fibrosis, and preserved left ventricular function, suggesting that increased oxidative stress may account for the changes in Ca^2+^ handling, myocardial dysfunction, and inflammation in *mdx* hearts [145]. Ca^2+^-induced Ca^2+^ release (CICR) and mitochondria-derived ROS generation exhibit a high degree of positive feedback, and CICR and elevated intracellular Ca^2+^ favor mitochondrial ROS production in dystrophin-deficient isolated cardiomyocytes [116]. Altered mitochondrial energy production is one of the first pathophysiological changes in the *mdx* heart, as precardiomyopathic *mdx* mice (10 to 12 weeks of age) in one study showed a slight shift in energy consumption from the normally utilized fatty acids to a higher usage of carbohydrates [146]. These early mitochondrial metabolic abnormalities in the subclinical stage are likely to compromise energy production and predispose these hearts to contractile dysfunction and cadiomyocyte membrane damage, thereby potentially contributing to the ultimate progression toward overt cardiomyopathy [146]. Increased intracellular Ca^2+^ levels independently increase mitochondrial membrane permeability through the mitochondrial permeability transition pore (PTP) [147]. The PTP is a voltage-sensitive channel, and an increase in the mitochondrial Ca^2+^ concentration induces the opening of PTP, leading to mitochondrial swelling and necrosis [147]. The NO-cGMP pathway is involved in PTP opening, as sildenafil attenuates stress-induced opening of PTP in association with reduced mitochondrial Ca^2+^ uptake in the dystrophin-deficient heart [148]. 

#### 3.2.4. Extracellular Matrix Remodeling and Myocardial Fibrosis

Death of cardiomyocytes is followed by focal inflammatory cascade within the necrotic areas in the myocardium. Increased inflammatory cells are not infrequently seen in ventricular myocardium of *mdx* mice and DMD patients, which precipitates myocardial fibrosis and an early deterioration of heart function [136,149]. Enhanced inflammation has been demonstrated in dystrophin-deficient myocardium in the mouse model with failing heart, mediated by dysregulation of metalloproteinase (MMP)-2 and MMP-9 [150,151]. Suppression of the inflammatory process by inhibition of nuclear factor κB (NF-κB) improved cardiac contractile function in utrophin/dystrophin-deficient mice [152], Once macrophages have removed the dead myocyte debris, fibroblasts then invade the damaged area and form a fibrocollagenous scar tissue resulting in the deposition of fibrotic tissue in the wall of ventricular myocardium [153,154]. This process, myocardial fibrosis, is characterized by enhanced production and deposition of extracellular matrix proteins by myofibroblasts, mediated mainly by transforming growth factor-β1 (TGF-β1) [155,156], which is activated by the renin-angiotensin system (RAS) [157]. TGF-β1 expression levels have been shown to correlate with muscle fibrosis in muscle biopsies from patients with DMD [158]. Up-regulation of connective tissue growth factor (CTGF) by TGF-β has been described in myocardium in both DMD patients [159] and *mdx* mice [160]. Whereas increased deposition of various extracellular matrix proteins was seen in the fibrotic myocardium, disintegration of basal lamina structure and cytoskeletal network was noted in the dystrophin-deficient heart, as presented by a drastic decrease in laminin, nidogen, and annexin [161]. Laminin is an essential component of basement membrane and critical for the structural integrity of the extracellular matrix, which forms a complex with nidogen [161]. Annexin plays a central role in maintenance of cytoskeleton and extracellular matrix [162] and Ca^2+^ handling in the heart [163]. 

### 3.3. Other Associated Pathological Mechanisms

As in some cases of heart disease and heart failure, certain epigenetic factors, including chromatin remodeling, DNA methylation, histone modification, and RNA-based mechanism (microRNAs), are known to participate in the final phenotype of dystrophinopathies in addition to the above-mentioned secondary cellular responses [164,165,166,167]. Other factors, post-translational modification and telomere dysfunction, are also known to influence a final phenotype independent of original genotype in the development of dystrophic hearts. Furthermore, genetic modifiers may influence the phenotype [168,169]. None of these factors function independently, but rather mediate complex interactions with Ca^2+^ homeostasis, NO-pathways, and mitochondrial function and ROS production to affect final phenotype of dystrophinopathies. 

#### 3.3.1. Epigenetic Factors

Compared with studies in skeletal muscle, studies regarding epigenetic regulation of dystrophic heart are limited. Nanni at al. demonstrated that the expression of nuclear pore protein Nup153 is up-regulated in dystrophin-deficient myocardium and that Nup153 regulates certain gene expression involved in cardiac remodeling and voltage-gated Ca^2+^ channels in response to NO and oxidative stress [170]. Nup153 is one of the nucleoporins that span the nuclear envelope, control nucleus-cytoplasm transport, and are considered as important regulators of gene expression and chromatic structure [171]. As discussed earlier, NO deficiency in association with mitochondrial dysfunction and increased ROS is a characteristic feature of dystrophin-deficient myocardium. Nup153 is a mediator of NO-altered signaling driving epigenetic alterations, contributing the development of dystrophic cardiac dysfunction and remodeling [170]. 

Histone acetylation occurs at the lysine residues of the histone tails, resulting in decondensation of the chromatin structure and acting as binding sites for bromodomain proteins and transcriptional activators, eventually leading to transcriptional activation [165]. Dystrophin deficiency leads to deregulated histone deacetylase (HDAC) activity, which disrupts downstream networks and can be restored by HDAC inhibitors in the skeletal muscle [172]. The molecular connection between DGC and chromatin has been described in muscle cells, as NO signaling regulates HDAC2 activity by *S*-nytrosylation, which inhibits HDAC2-mediated gene repression [173]. Intervention targeting histone deacetylases (HDACs), by direct inhibitors or by reconstituting the NO signaling, was proven effective in ameliorating DMD phenotype [173,174]. HDAC inhibition has been shown to rescue ischemia-induced DMD-signature alteration and improve phenotype in *mdx* mice, suggesting that DMD-signature miRNAs may serve as useful markers for therapeutic purposes [175]. However, the direct involvement of HDAC in the dystrophin-deficient heart has not been demonstrated thus far. 

Micro-RNAs (miRNAs) are small noncoding RNA molecules that regulate the stability and/or translational efficiency of target mRNAs [176]. Many miRNAs are known to play critical roles in the pathogenesis and progression of heart failure [177,178]. Eisenberg et al. identified a series of miRNAs that are regulated in almost all myopathies and those specifically in DMD during the degenerative process of skeletal muscle [179]. There is a distinct pattern of dysregulation of miRNAs in DMD, which may become potential therapeutic targets for future clinical applications [179]. In a damage/regeneration mouse model in dystrophin deficiency, DMD-signature miRNAs were detected and divided into three classes: (1) regenerative miRNAs; (2) degenerative miRNAs; and (3) inflammatory miRNAs [175]. In the dystrophic heart, certain miRNAs have been identified to play an important role in disease progression. Downregulation of miR-1, miR-133a, and miR-208 was noted in cardiac progenitor cells (CPCs) of Golden Retriever muscle dystrophy (GRMD) dogs with dysfunctional heart, suggesting decreased cardiac commitment of CPCs in GRMD [180]. Drastic downregulation of miR-448 in the heart of *mdx* mice was shown to be responsible for oxidative stress-induced cardiac remodeling [181]. In the human dystrophic heart, increased serum miR-222, miR-26a, and miR-378a-5p were shown to indicate the presence of myocardial scars [182]. Further investigation will be required to delineate a complex network among multiple miRNAs in the development of dystrophic cardiomyopathy. 

#### 3.3.2. Post-Translational Modification

The cellular pathology of the failing heart often shows impaired intracellular Ca^2+^ homeostasis, as described earlier. Ryanodine receptor (RyR), a Ca^2+^-binding protein on the sarcoplasmic reticulum (SR), regulates CICR from the SR. Post-translational modification of RyR enhancing Ca^2+^ sensitivity and CICR is shown to primarily drive functional deterioration in dystrophic heart in combination with increased oxidative stress [109]. In dystrophin deficiency, connexin 43 (Cx43), the most abundant cardiac gap junction protein localized predominantly in the intercalated disc, is mostly lateralized, which causes alteration in electrical propagation in the myocardium in association with oxidative stress [183]. Gap junction remodeling with lateralization of Cx43 is also seen in myocytes in the epicardial border zone after experimental myocardial infarction [184] and other cardiomyopathies [185]. Post-translational modification of N^ε^-lysine acetylation of Cx43 induces the dystrophic heart to lose normal regulatory control of Cx43 localization in intercalated discs, which affects cardiac rhythm [108]. Disorganized Cx43 expression pattern in the cardiomyocytes significantly contributes to arrhythmogenesis and increases the risk of sudden death [186]. 

#### 3.3.3. Telomere Dysfunction

Telomere shortening occurs during aging and activates senescence and apoptotic programs that compromise the function of organs with high rates of proliferation and turnover [187]. Long telomeres protect *mdx* mice from developing cardiomyopathy, unlike DMD patients who frequently develop DCM in their mid-teens [187]. Mourkioti et al. proposed that the demands of contraction in the absence of dystrophin with increased oxidative stress combine to accelerate telomere erosion, resulting in telomere shortening and cardiac failure [106]. Telomere shortening worsens myocardial function by impairing mitochondrial biogenesis. Cellular respiration and ATP output are therefore severely compromised, suggesting a link between telomere shortening and mitochondrial dysfunction in the etiology of DCM in DMD [107]. 

#### 3.3.4. Genetic Modifiers

A modifier gene is a genetic locus that enhances or suppresses the outcome of the primary disease-causing mutation and may affect different aspect of the disease, such as age at onset, severity, or duration [168]. The modifier genes on DMD were primarily studied in the skeletal muscle [168,169]. Osteoponin (secreted phosphoprotein 1 or SPP1) was found to promote fibrosis in dystrophic mouse muscle by modulating TGF-β pathway [188]. Polymorphism in latent TGF-β binding protein 4 (LTBP4) influenced the age of loss of ambulation in DMD patients [189]. Certain polymorphisms in SPP1 and LTBP4 were shown to have protective effects against DCM in DMD patients when treated with corticosteroid [190]. Annexin A6 was also identified as a modifier of muscular dystrophy that plays a role in stabilizing and repairing sarcolemma after injury [191]. Right ventricular hypertrophy, a frequent association with respiratory failure in DMD patients, may be, in part, a direct effect of altered annexin A6 expression, as annexin A6 is highly expressed in both skeletal muscle and myocardium [191]. 

## 4. Conclusions

**Figure 2:**
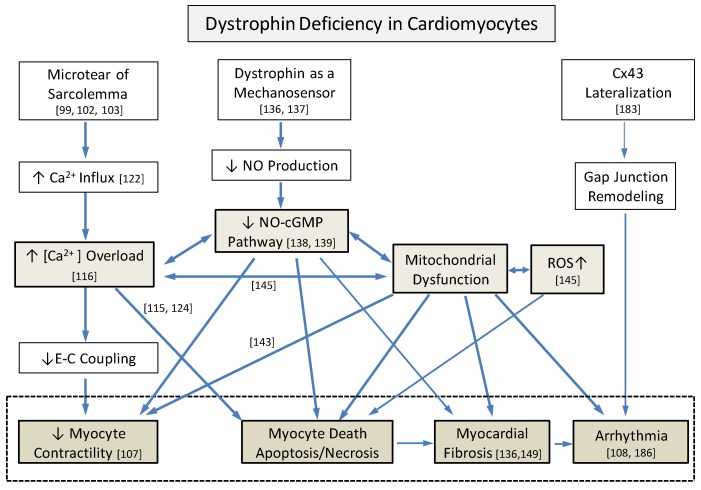
Complex underlying pathological interactions among primary structural vulnerability and secondary cellular responses that induce dystrophic hearts from dystrophin deficiency. Three major processes, including intracellular Ca^2+^ overload, decreased NO-cGMP pathways, and mitochondrial dysfunction with increased reactive oxygen species (ROS), interact closely. Numbers indicate references. Cx43: connexin 43, CICR: Ca^2+^-induced Ca^2+^- response, SACs: stretch-activated channels, TRPC: transient receptor potential cation channels, NCX: Na^+^-Ca^2+^ exchanger, VGCC: voltage-gated Ca^2+^ channels, and E-C coupling: excitation-contraction coupling.

Dystrophinopathies are a group of genetic disorders caused by mutations in dystrophin. These mutations result in diverse clinical phenotypes involving skeletal muscles and myocardium with frequent involvement of other organ systems. Secondary cellular and tissue responses to dystrophin deficiency including abnormal intracellular Ca^2+^ homeostasis, altered NO signaling, and mitochondrial dysfunction and ROS production significantly influence final clinical phenotype (Figure 2). Dystrophin deficiency alters epigenetic and other secondary factors, which may also contribute to the final phenotype. Absence of clear genotype-phenotype correlation in dystrophinopathies is the result of these multi-layered complex pathobiological processes. With increased understanding of baseline pathogenesis, innovative therapeutic approaches to these lethal disorders have been proposed [192,193]. However, when compared with the skeletal muscle, pathobiology of dystrophin-deficient myocardium has not been fully investigated. Dystrophinopathies are an excellent human model to understand heterogeneity and complexity of genetic disorders. Multiple medical treatments may be considered upon targeting each pathological process.
